# Cytosolic Phospholipase A2 Modulates TLR2 Signaling in Synoviocytes

**DOI:** 10.1371/journal.pone.0119088

**Published:** 2015-04-20

**Authors:** Randi M. Sommerfelt, Astrid J. Feuerherm, Trine Skuland, Berit Johansen

**Affiliations:** Department of Biology, Lipid Signaling Group, Norwegian University of Science and Technology, Trondheim, Norway; Universidade Federal do Rio de Janeiro, BRAZIL

## Abstract

Rheumatoid arthritis (RA) is an autoimmune disease characterized by chronic synovitis leading to destruction of cartilage and bone. PLA2 enzymes are key players in inflammation regulating the release of unsaturated fatty acids such as arachidonic acid (AA), a precursor of pro-inflammatory eicosanoids. Several lines of evidence point to toll-like receptors (TLRs) as drivers of synovitis and joint destruction in RA. However, few studies have addressed the implication of PLA2 activity downstream TLR activation in the synovium. Here, we aimed to characterize PLA2 enzyme involvement in TLR2-induced signaling in synovial fibroblast-like cells. TLRs1-7 and a range of sPLA2, iPLA2 and cPLA2 enzymes were found to be transcriptionally expressed in cultured synoviocytes. Activation of TLR2/1 and TLR2/6 led to phosphorylation of cPLA2α at Ser^505^, and induced AA release and PGE_2_ production; effects that were attenuated by cPLA2α inhibitors. In contrast, sPLA2 inhibitors did not affect AA or PGE_2_ release. cPLA2α inhibitors furthermore attenuated TLR-induced expression of IL-6, IL-8 and COX2. COX1/2 inhibitors attenuated TLR2/6-induced IL-6 transcription and protein production comparable to cPLA2α inhibition. Moreover, exogenously PGE_2_ added alone induced IL-6 production and completely rescued IL-6 transcription when added simultaneously with FSL-1 in the presence of a cPLA2α inhibitor. Our results demonstrate for the first time that cPLA2α is involved in TLR2/1- and TLR2/6-induced AA release, PGE_2_ production and pro-inflammatory cytokine expression in synoviocytes, possibly through COX/PGE_2_-dependent pathways. These findings expand our understanding of cPLA2α as a modulator of inflammatory molecular mechanisms in chronic diseases such as RA.

## Introduction

Rheumatoid arthritis (RA) is a complex systemic inflammatory disease characterized by chronic synovitis and irreversible destruction of cartilage and bone. The aetiology of RA is unclear, but genetic, epigenetic and environmental factors are involved in triggering and/or exacerbating RA synovitis [1, 2]. Fibroblasts are believed to play an important role in chronic inflammation [3], and RA fibroblast-like synoviocytes (FLS) actively promote inflammation and joint destruction [4].

Lipid metabolites derived from the unsaturated Ω6 fatty acid arachidonic acid (AA) play pivotal roles in inflammation [5]. The eicosanoid prostaglandin E2 (PGE_2_) is metabolized from AA by the cyclooxygenase (COX) enzymatic pathway, and is a key regulator of immunopathology and chronic inflammation [6]. PGE_2_ is abundantly detected in synovial fluid of arthritic joints [7], and the effective symptomatic relief in RA patients by non-steroid anti-inflammatory drugs (NSAIDs) targeting the COX enzymes is in large part due to decreased PGE_2_ synthesis [8]. Phospholipase A2 (PLA2) enzymes act to hydrolyze membrane phospholipids at the *sn*-2 position to liberate unsaturated fatty acids from cellular membranes, and are thus important regulators of lipid signaling [9]. To date, the PLA2 enzyme family includes 16 groups comprising more than 30 members. Based on their biochemical properties, PLA2 enzymes are subdivided into distinct types; Ca^2+^-dependent secretory PLA2s (sPLA2s), Ca^2+^-dependent cytosolic PLA2s (cPLA2s), Ca^2+^-independent cytosolic PLA2s (iPLA2s), platelet-activating factor acetyl hydrolases (PAF-AHs), lysosomal PLA2 and adipose-PLA2 [9]. Cytosolic group IVa PLA2 (cPLA2α) is activated by intracellular calcium and by phosphorylation in response to various extracellular stimuli. cPLA2α is selective for AA-containing acyl chains *in vitro* [10], and is considered a central enzyme in AA-derived eicosanoid production [9]. sPLA2 and iPLA2 also contribute to AA release, although they do not display the same acyl chain specificity as cPLA2α [11, 12]. Due to its arachidonyl selectivity, cPLA2α is believed to play a key role in inflammatory disease, a view supported by the findings that cPLA2α-deficient mouse models are resistant to various inflammatory diseases including asthma, pulmonary fibrosis and CIA-induced arthritis [13–16]. Moreover, inhibitors targeting cPLA2α decelerate disease progression in CIA mice [17, 18]. However, through which mechanisms cPLA2α-deficiency or inhibition prevent disease progression is not fully understood.

Toll-like receptors (TLRs) are pattern recognition receptors (PRRs), constituting a major part of the innate immune system sensing pathogen associated molecular patterns (PAMPs) on invading pathogens [19]. Moreover, TLRs can induce non-infectious inflammation by sensing endogenous molecules released in response to tissue damage or necrosis (damage associated molecular patterns, DAMPs), and elevated TLR activation is associated with several inflammatory, autoimmune and non-infectious diseases including RA [20]. The TLR2 family of receptors (TLR1, TLR2, TLR6) is located on the cell surface. TLR2 dimerizes with TLR1 or TLR6 to recognize a range of PAMPs and DAMPs [20], of which several, including bacterial lipoproteins [2] and heat-shock proteins [21, 22], are detected in RA joints. In FLS from RA patients, TLRs including TLR2 and 6 levels are significantly elevated compared to patients with non-inflammatory arthritis [23], and TLR2 is found in excess at sites of pannus invasion and cartilage and bone erosion [24]. Accordingly, TLR2 activation is believed to play a role in chronic inflammation and joint destruction in RA.

TLR2 ligands are reported to activate PLA2 in human leukocytes and murine macrophages [25, 26]. However, interactions between PLA2 enzymes and TLR2 signaling in synoviocytes are hitherto not well described. Here, we propose that cPLA2α is a major regulator of TLR2-induced AA release and PGE_2_ production in human synoviocytes. In contrast, sPLA2 involvement was not found. Furthermore, we demonstrate that cPLA2α inhibition attenuates TLR2-induced expression of inflammatory cytokines, suggesting a regulatory role of cPLA2α in synovial TLR responses.

## Materials and Methods

### Reagents

PBS was from Oxoid. DNAse- and RNAse-free water was from VWR. Recombinant human TNF and IL-6 ELISA Duoset were from R&D systems. Quantitect primer assays for TLR1-7 and 18S were from Qiagen. QuantiTect Reverse Transcription kit, RNeasy minikit, Leupeptin, pepstatin and LightCycler 480 SYBR Green I Master mix were from Roche Molecular Biochemicals. RNA*later* was from Life technologies. FSL-1 and Pam_3_CSK_4_ were from Invivogen. [^3^H]-arachidonic acid ([^3^H]-AA), and liquid scintillation cocktail Ultima Gold were from NEN Perkin Elmer. AVX002 and Inhibitor 28 were provided by Avexxin AS (Trondheim, Norway). Arachidonyl trifluoromethyl ketone (AACOCF3, ATK) was from Enzo Life Sciences. Varespladib (LY315920) was from Selleckchem. CAY10502, CAY10590 and PGE_2_ ELISA kit were from Cayman Chemicals. Phospho-cPLA2 (Ser505) antibody was from Cell Signal Technology. α-tubulin antibody was from Santa Cruz Biotechnology. Polyclonal goat anti-mouse immunoglobulins horse radish peroxidase-conjugated secondary antibody was from Dako. Hybond-C nitrocellulose membranes were from GE healthcare. NuPAGE gel system (10% Bis-Tris gel) was from Invitrogen. Hybond-C nitrocellulose membranes were from GE healthcare. SuperSignal West Femto Maximum Sensivity substrate was from Thermo Scientific. All other reagents were from Sigma-Aldrich.

### Cell culture

The human synovial sarcoma derived cell line SW982 (ATCC, London, UK) was maintained at 37°C with 10% CO_2_ in a humidified atmosphere in Dulbecco's Modified Eagle Medium (DMEM) supplemented with 10% FBS, 0.1 mg/mL gentamicin and 0.3 mg/mL L-glutamine. Experiments were performed at 3 days post-confluence. Prior to experimental treatment, cells were serum-deprived in serum-free DMEM overnight, and all experiments were performed in serum-free DMEM. Inhibitors used were applied 2 hours before stimulation with FSL-1, Pam_3_CSK_4_ or TNF.

### [^3^H]-arachidonic acid release assay

SW982 cells were labeled for 18 hours with [^3^H]AA in serum-free DMEM and washed in PBS prior to experimental treatment. Release of [^3^H]AA was analyzed in triplicates as previously described [27]. In short, cells were incubated for the desired period of time in the absence or presence of chemical inhibitors prior to stimulation with FSL-1 or Pam_3_CSK_4_. Supernatants were removed and cleared of detached cells or cell debris by centrifugation. Cells attached in the wells were dissolved in 1M NaOH and all samples were assayed for fatty acid release by scintillation counting. Results are shown as released [^3^H]AA in supernatants relative to total [^3^H]AA incorporated into the cells. All experiments were performed in triplicates.

### PGE_2_ and IL6 enzyme-linked immunosorbent assays (ELISA)

Serum-deprived SW982 cells were treated with FSL-1 or Pam_3_CSK_4_ for 24 hours in the absence or presence of chemical inhibitors. Supernatants were harvested, and PGE_2_ and IL-6 levels were analyzed by ELISA according to the respective kit protocols. The read-out was carried out with a Multiscan plate reader (Ascent Labsystems). The corresponding Ascent software for Multiscan, Version 2.4.1 was used to obtain the raw data. A 4-parameter logistic curve fitting was used to analyze the data.

### Real-time reverse-transcription polymerase chain reaction (qPCR)

One sample of post-natal full-term placenta was collected from St Olavs hospital (Trondheim, Norway). Small pieces of placenta were immediately placed in RNA*later* and stored at -80°C. No further permissions are required from Regional Committees for Medical and Health Research Ethics (REC, Norway) for use of anonymized biological material in the technical and methodological work presented in current publication. Total RNA from placental and SW982 cells was isolated using RNeasy minikit according to kit protocol. RNA concentrations and integrity was monitored by NanoDrop spectrophotometric measurement (NanoDrop Technologies Inc.) and total RNA (1 μg) was reverse transcribed using the QuantiTect Reverse Transcription kit following the recommended protocol. qPCR analyses were carried out using the Lightcycler 480 system. Primers for PLA2 subgroups, IL-6, IL-8, COX2 and GAPDH were designed with Primer 3 software (Whitehead Institute for Biomedical Research, Cambridge, MA, USA). Primer sequences or Qiagen reference sequence for all primers applied are listed in Tables 1 and 2, respectively. Dissociation curve analysis for each SYBR Green primer pair and reaction was performed to verify specific amplification. Primer amplification efficiencies and Cq-values were calculated by the LinRegPCR software [28] and fold changes in mRNA expression were analyzed by the QbasePLUS software (Biogazelle) using GAPDH or 18S as reference gene.

**Table 1:** Overview of qPCR primers.

Gene	Forward primer	Reverse primer	Product size (basepairs)
PLA2G2A	aaggaagccgcactcagtta	ttgcacaggtgattctgctc	186
PLA2G2D	gatggtcaagcaagtgactgg	gagcagtggatgttcccctg	213
PLA2G4A	catgcccagacctacgattt	cccaatatggctaccacagg	163
PLA2G4C	tgccggagtctcatttgtcc	gggtgaactcgaaccaggtc	141
PLA2G4D	cgtcagatcgcccagaaaac	ccaggaaggatgtctgtgtgt	177
PLA2G4F	gagttggaggctcagaccag	cagagaggtcaaagccaagg	178
PLA2G5	gccaaagagaaccccagag	gccgtagaagccgtagtttg	154
PLA2G6A	ttatgctgtccagggtgaca	gagaacttcatggccgagtg	216
PLA2G6B	tggagccatgcattttatga	gacatgtggggtttcttgc	100
PLA2G6C	ctggaacctgtgttggacct	cggtgatatctgtggtcacg	146
PLA2G6D	cgtggatgccttggtatgttc	aagggtacgttgtcactcact	111
PLA2G6F	taacgcccggttatgacttc	tgctggctaggaggacctta	186
PLA2G7A	attgacctggcatctcatggg	ccaagacttgtcccctatttctg	117
PLA2G10	cctggcagtgcgtcaatca	tgtactcagtttgggctaagca	114
PLA2G12A	ggatgtggctctccactgtt	tgccacaggtctcatagcac	100
IL-6	tgtgtgaaagcagcaaagag	gcaagtctcctcattgaatcc	104
IL-8	gacatactccaaacctttccac	cttctccacaaccctctgc	160
COX2	ggggatcagggatgaacttt	tggctacaaaagctgggaag	172
GAPDH	catcaagaaggtggtgaagcag	tgtagccaaattcgttgtcatacc	191

**Table 2:** Overview of qPCR Primer Assays (Qiagen).

Gene	Qiagen RefSeq accession	Product size (basepairs)
TLR1	NM_003263.3	189
TLR2	NM_003264.3	154
TLR3	NM_003265.2	97
TLR4	NM_138554.3	68
TLR5	NM_003268.5	136
TLR6	NM_006068.4	151
TLR7	NM_016562.3	195
18S rRNA	X03205.1	100

### Immunoblotting

Following FSL-1 or Pam_3_CSK_4_ treatment for indicated times with or without chemical inhibitors, cells were washed in PBS and lysed in buffer containing 50mM Tris pH 7.5, 150 mM NaCl, 10% glycerol, 0.5% Triton-X-100, 2mM EDTA, 40 mM β-glycerophosphate, 100 mM NaF, 200 μM Na_3_VO_4_, 10 μg leupeptin, 1 μM pepstatin and 1mM phenylmethylsulfonyl fluoride. Lysates were cleared by centrifugation, separated by SDS-PAGE and transferred to Hybond-C nitrocellulose membranes by electrophoresis using the NuPAGE gel system (10% Bis-Tris gel). Membranes were blocked in Tris-buffered saline containing 0.1% Tween 20 and 5% non-fat dry milk for 1 hour at room temperature before incubation with the primary antibodies overnight at 4°C. Following washing three times with Tris-buffered saline containing 20% Tween 20, target proteins were detected by horseradish peroxidase-conjugated secondary antibody (1 hour at room temperature) and SuperSignal West Femto Maximum Sensivity substrate. Blots were analyzed by the BioRad Image Lab software, and target protein band intensities were normalized to α-tubulin.

### Statistical analysis

For AA release and ELISA analysis, statistical analyses were performed by one-way analysis of variance (ANOVA) in conjunction with Bonferroni's post hoc test for multiple comparison (IBM SPSS Statistics 20 software) or Student t-test when appropriate. For qPCR data, statistical analyses were performed by the QbasePLUS software (Biogazelle). Differences were considered significant at p ≤ 0.05.

## Results

### Synoviocytes express cPLA2, sPLA2 and iPLA2 enzymes, of which cPLA2α is regulated by TLR2 ligands

**Fig 1:**
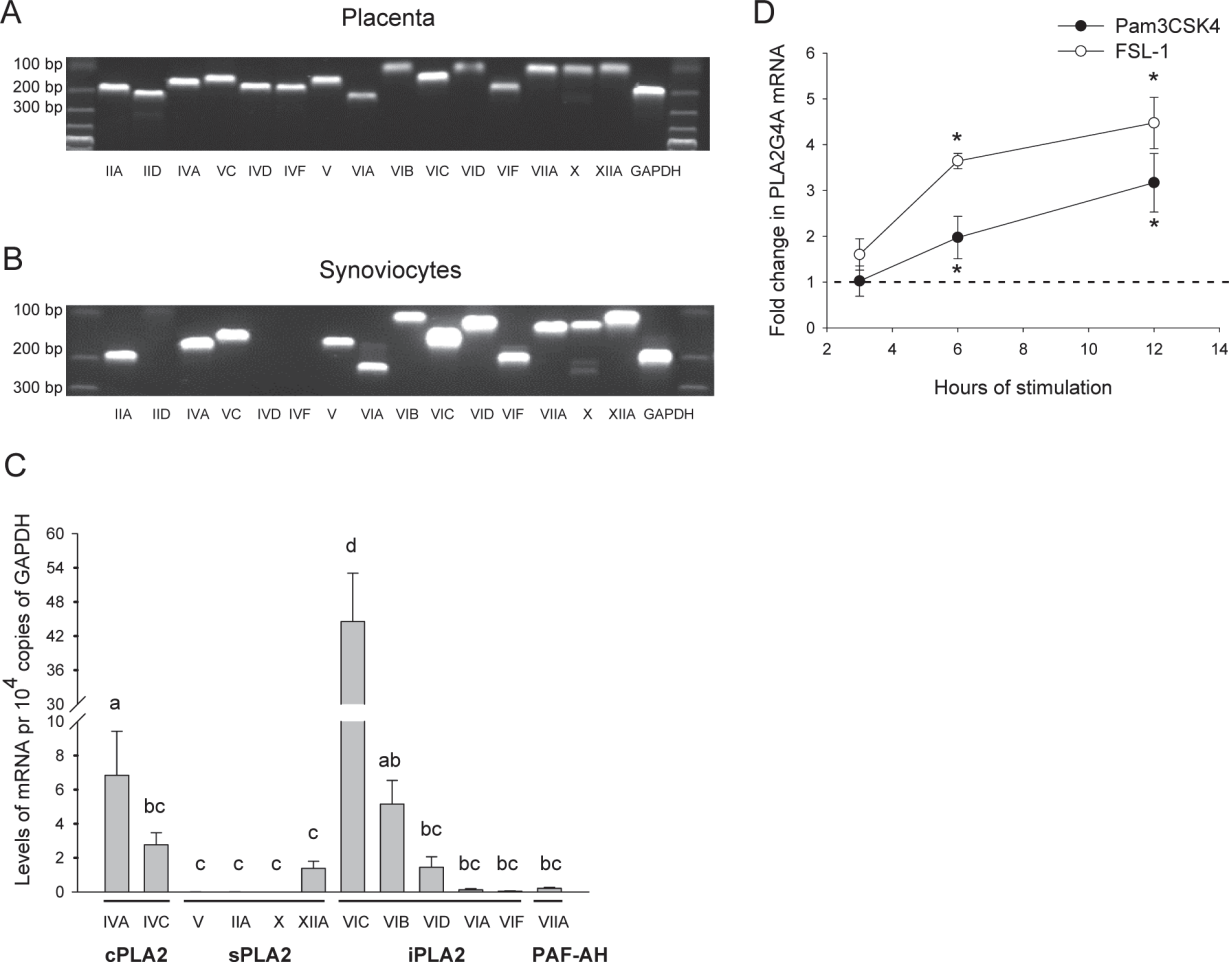
SW982 synoviocytes express a variety of PLA2 enzymes. Transcriptional expression of PLA2 enzymes in placenta and unstimulated synoviocytes was determined by qPCR as described in the Methods section. PCR product specificity from placenta (**A**) and synoviocytes (**B**) were analyzed by gel electrophoresis according to fragment size. In unstimulated synoviocytes, expression profile of the detected PLA2 subgroups was calculated in reciprocal comparison to GAPDH (**C**). Data shown are mean ± SD of 3 independent experiments, results are considered significant at p ≤ 0.05. Means sharing a common alphabetical symbol did not differ significantly. Synoviocytes were treated with FSL-1 (100 ng/mL) or Pam_3_CSK_4_ (200 ng/mL) for indicated time and transcriptional expression of PLA2G4A was determined by qPCR as described in the Methods section (**D)**. Results are concidered significantly upregulated (p* ≤ 0.05) when compared to untreated control values.

Different human PLA2 isoforms are detected in various tissues and cell types. In RA synovium, cPLA2α and several sPLA2s are detected, and mRNA expression correlate to the presence of protein [29, 30]. PLA2 transcriptional expression can further be increased by pro-inflammatory cytokines in cultured synovial cells [29, 30]. Cellular enzymatic activity of PLA2 enzymes can be induced by various inflammatory stimuli including TLR ligands [25, 26], but the downstream effects of PLA2 enzyme activity in synoviocyte TLR signaling is not well known. To further characterize the repertoire of PLA2 enzymes expressed in synoviocytes, we investigated the transcriptional expression of 16 different PLA2 genes by qPCR. PLA2 subgroups GIIA, GIID, GIVA, GIVD, GIVF, GV, GVIA, GVIB, GVIC, GVIF, GVIIA, GX and GXIIA were first transcriptionally detected in placental tissue samples (Fig 1A) to ascertain primer function and specificity as placenta is reported to express a large repertoire of PLA2s including sPLA2s (GIIA, GIID, GV), cPLA2s (GIVA, GIVD), iPLA2 (GVI) and PLA2GVII [31–34]. All PLA2 subgroups were also detected in synoviocytes, except for GIID, GIVD and GIVF (Fig 1B), with the relative reciprocal expression profiles presented in Fig 1C. Transcripts for cPLA2 enzymes GIVA and GIVC were detected at relatively high levels. Of the sPLA2s, GXIIA was the most highly expressed while GIIA, GV and GX were detected at very low levels. Six subgroups of iPLA2s, and also one PAF-AH, GVIIA, were detected.

We next investigated if PLA2 enzyme mRNA levels were regulated in response to the TLR2/1 and TLR2/6 ligands Pam_3_CSK_4_ and FSL-1. We found that GIVA was significantly upregulated by the TLR2 ligands after 6 hours of stimulation, 2-fold by Pam_3_CSK_4_ and 4-fold by FSL-1 (Fig 1D). No significant change in mRNA expression was detected for any other PLA2 subgroups. Thus, SW982 synoviocytes express a repertoire of PLA2 enzymes, of which cPLA2α stand out as it is transcriptionally induced by activators of TLR2/1 and TLR2/6. Moreover, these results suggest activation of functional TLR1/2 and TLR2/6 dimers, leading to activation of gene transcription.

### Synoviocytes express a repertoire of TLRs

**Fig 2:**
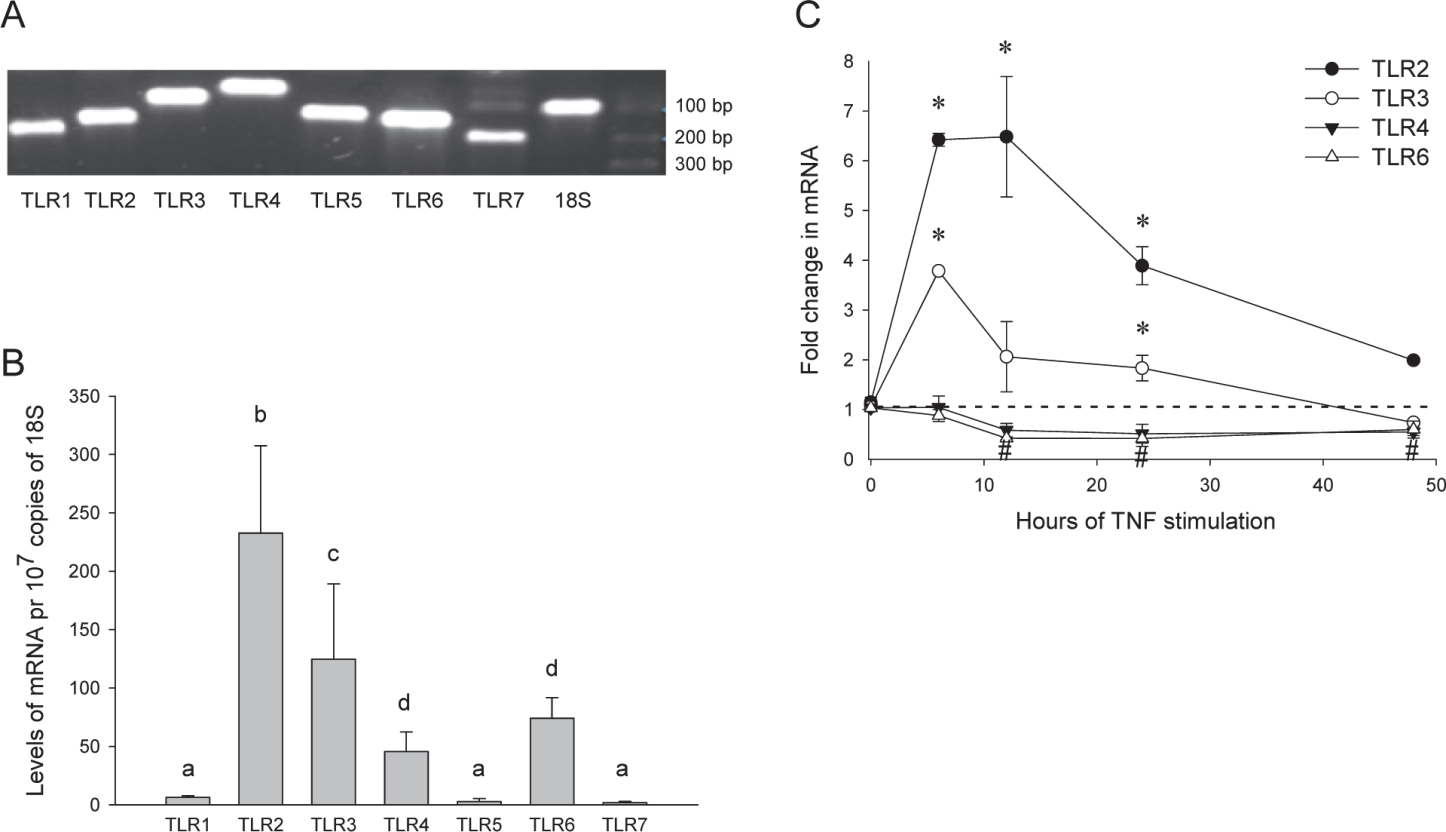
SW982 synoviocytes express TLRs 1–7 gene transcripts. Transcriptional expression of TLRs 1–7 in unstimulated synoviocytes was determined by qPCR as described in the Methods section. qPCR products in untreated cells were analyzed by gel electrophoresis **(A)**. Relative reciprocal mRNA expression levels normalized to 18S (mean ± SD from 3 independent experiments) (**B**). Differences are considered significant at p ≤ 0.05. Means sharing a common alphabetical symbol do not differ significantly. Cells were treated with TNF (10 ng/mL) for indicated time **(C**). Results shown are mean ± SD of three independent experiments with 18S as reference gene. Results are concidered significantly upregulated (p* ≤ 0.05) or downregulated (p^#^ ≤ 0.05) compared to untreated controls at each timepoint.

FLS in RA synovium express a range of TLRs, whose transcriptional expression correlate to immunohistochemical staining [23]. In FLS from RA joints, TLR2 and TLR4 are regulated by pro-inflammatory stimuli such as tumor necrosis factor (TNF), a central pro-inflammatory cytokine in RA pathogenesis [24]. We characterized the expression of TLRs1-7 in SW982 synoviocytes by qPCR. Transcripts for all seven TLR receptors were detected (Fig 2A), of which TLR2 was most abundantly expressed (Fig 2B). We next investigated whether TLR expression was regulated by TNF. TLR2 and TLR3 mRNA expression were induced in a time-dependent manner peaking at 6 hours (6- and 4-fold, respectively, Fig 2C). In contrast, TLR4 and TLR6 were time-dependently down-regulated by 50% in response to TNF after 12 hours (TLR6), 24 and 48 hours of stimulation (TLR4, TLR6). Thus, SW982 synoviocytes express TLRs 1–7, of which TLRs2, 3, 4 and 6 are transcriptionally regulated by TNF.

### TLR2 ligands induce cPLA2α phosphorylation at Ser^505^

**Fig 3:**
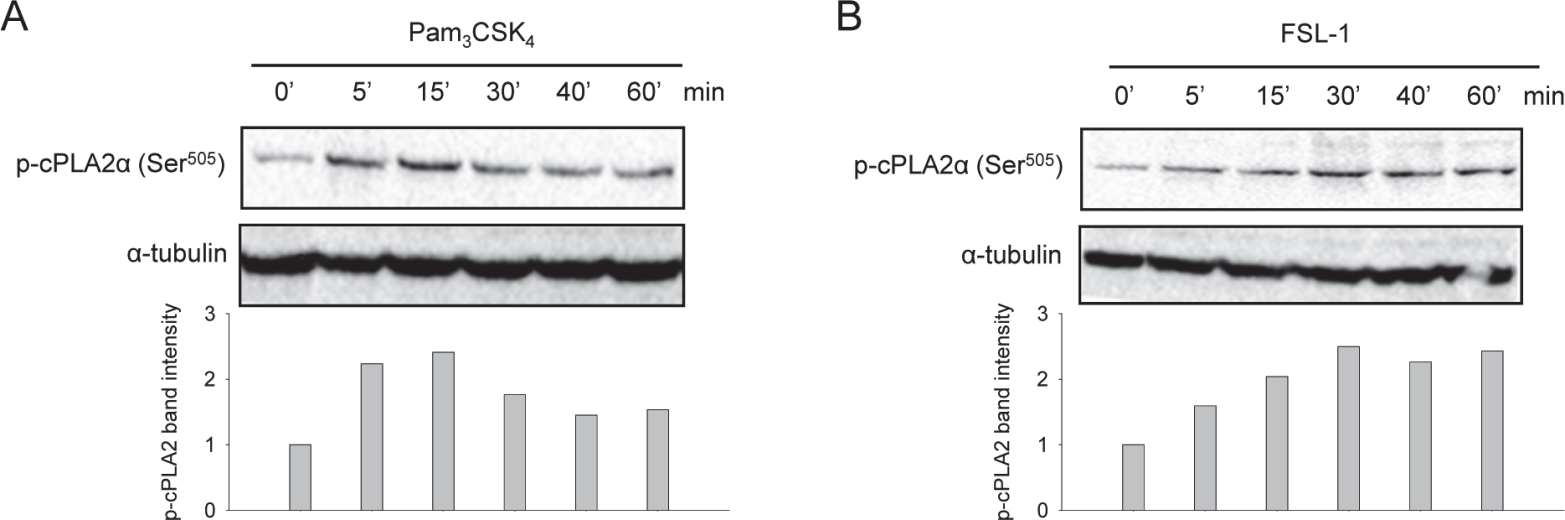
TLR2 ligands induce cPLA2α phosphorylation at Ser^505^. Synoviocytes were treated with Pam_3_CSK_4_ (200 ng/mL) **(A)** or FSL-1 (100 ng/mL) **(B)** for indicated periods of time. Cell lysates were prepared and analyzed by immunoblotting for activation of phospho-cPLA2α (Ser^505^) as described in the Methods section. Immunoblotting for α-tubulin was performed to assess protein loading. Results shown are one representative of three independent experiments.

In response to pro-inflammatory stimuli, cPLA2α can be activated by phosphorylation at Ser^505^ [9]. To evaluate TLR2-induced cPLA2α activation in synoviocytes, we analyzed cPLA2α phosphorylation at Ser^505^ by immunoblotting. Both Pam_3_CSK_4_ and FSL-1 rapidly evoked time-dependent cPLA2α phosphorylation, evident after 5 minutes of stimulation (Fig 3). Pam_3_CSK_4_-induced cPLA2α phosphorylation peaked after 15 min, to decrease after 30 min (Fig 3A). In comparison, the FSL-1 response was slower; FSL-1 induced a maximum phosphorylation after 30 minutes that persisted throughout 60 min of stimulation (Fig 3B). These results suggest that Pam_3_CSK_4_ and FSL-1 both activate cPLA2α.

### Pam_3_CSK_4_- and FSL-1-induced AA release and PGE_2_ production depend on cPLA2α activity, not sPLA2 activity

**Fig 4:**
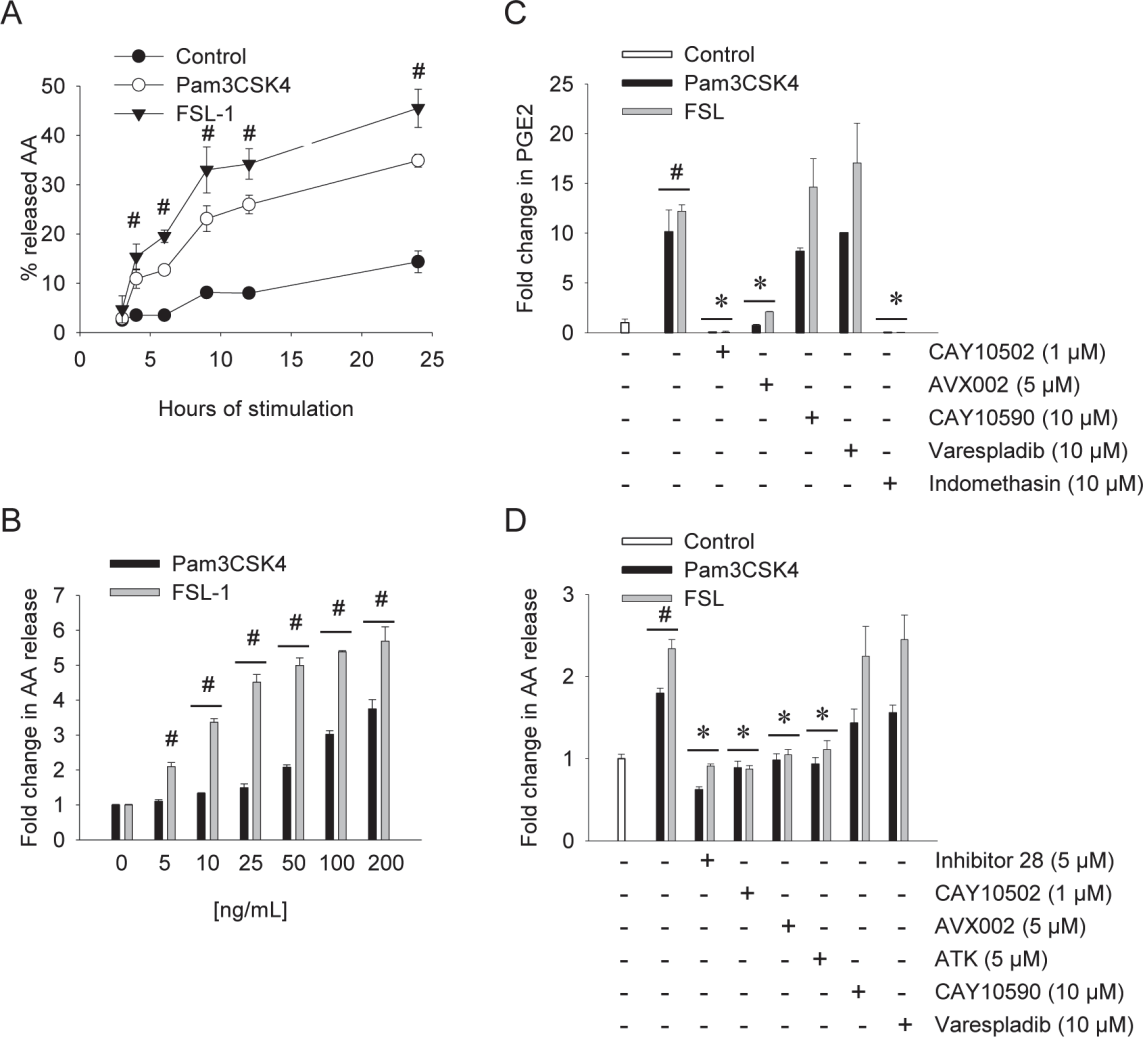
TLR2/1- and TLR2/6-induced AA release and PGE_2_ production depend on cPLA2α, not sPLA2 activity. Synoviocytes were treated with Pam_3_CSK_4_ or FSL-1 to determine AA release for indicated times (200 ng/mL and 100 ng/mL, respectively) **(A)**, or in indicated concentrations **(B)** for 4 hours. Release of AA was measured as described in the Methods section. Data presented are mean ± SD of one representative of at least 3 independent experiments, and statistical significance is indicated by p^#^ ≤ 0.05 when compared to untreated control samples. Cells were treated with inhibitors for cPLA2α (5 μM Inhibitor 28, 1 μM CAY10502, 5 μM AVX002, 5 μM ATK); sPLA2 (10 μM CAY10590, 10 μM Varespladib) or COX1/2 (10 μM Indomethasin) for 2 hours followed by FSL-1 (100 ng/mL) or Pam_3_CSK_4_ (200 ng/mL) for 24 hours (**C**) or 4 hours (**D**). Release of PGE_2_ (**C**) or AA (**D**) was measured by ELISA and AA release assay, respectively, as described in the Methods section. Results shown are mean ± SD of three independent experiments. Stimulated controls are compared to untreated controls whereas inhibitor-treated samples are compared to stimulated controls. Statistical significance is indicated by p^#^ ≤ 0.001 when compared to untreated control samples; p* ≤ 0.001 when compared to the respective ligand-stimulated samples **(C)**; p^#^ ≤ 0.05 when compared to untreated control samples; p* ≤ 0.05 when compared to the respective TLR ligand-treated samples **(D)**.

AA release and eicosanoid production induced by various TLR2 ligands is mediated by cPLA2α activity in human leukocytes [25]. In murine macrophages and mast cells, TLR-induced cPLA2α activity is modulated by sPLA2 GV [26, 35]. However, little is known concerning TLR-induced PLA2 activity and lipid metabolism in synoviocytes. Having found that FSL-1 and Pam_3_CSK_4_ stimulation lead to cPLA2α phosphorylation, we next investigated their effect on AA release and PGE_2_ production. In a time-dependent manner, AA release was significantly increased following 4 hours stimulation (3-fold by Pam_3_CSK_4_ and 4-fold by FSL-1), and persisted through 24 hours (Fig 4A). In dose-response experiments, FSL-1 was a more potent inducer of AA release than Pam_3_CSK_4_ in most concentrations tested (p < 0.001) (Fig 4B). Subsequent PGE_2_ production was increased 10-12-fold by both ligands with no significant difference between the two (Fig 4C). Thus, the increased PGE_2_ levels in response to TLR2 ligands correspond to the increased availability of AA.

To investigate the roles of sPLA2s and cPLA2α in TLR2-induced synoviocyte AA release and PGE_2_ production, we next analyzed the effects of synthetic inhibitors of cPLA2α (Inhibitor 28 [36], CAY10502 [37], AVX002 [38, 39], ATK [40],) or sPLA2 (CAY10590 [41], Varespladib [42]). In response to both TLR2 ligands, all cPLA2α inhibitors efficiently reduced AA release to untreated control level (Fig 4D). In contrast, sPLA2 inhibitors did not affect the release of AA (Fig 4D), as confirmed by dose-response (0.2–50 μM) and time course (4 and 24 hours) experiments (results not shown). Corresponding results were found in PGE_2_ production; inhibitors of cPLA2α potently attenuated PGE_2_ production in response to both Pam_3_CSK_4_ and FSL-1, whereas sPLA2 inhibitors did not (Fig 4C). Due to the lack of effect in synoviocytes, CAY10590 and Varespladib were evaluated in the keratinocyte cell line HaCaT known to express functional sPLA2 enzymes that contribute to AA release [43]. CAY10590 and Varespladib completely blocked IL-1β-induced AA release (144.2 ± 3.8% and 104.0 ±6.8%, respectively, results not shown) indicating that the sPLA2 inhibitors are indeed effective. In summary, these results suggest that cPLA2α has a more prominent role than sPLA2 enzymes in releasing AA for PGE_2_ production downstream TLR2 activation.

### TLR2-induced gene transcription and protein production involve cPLA2α activity

**Fig 5:**
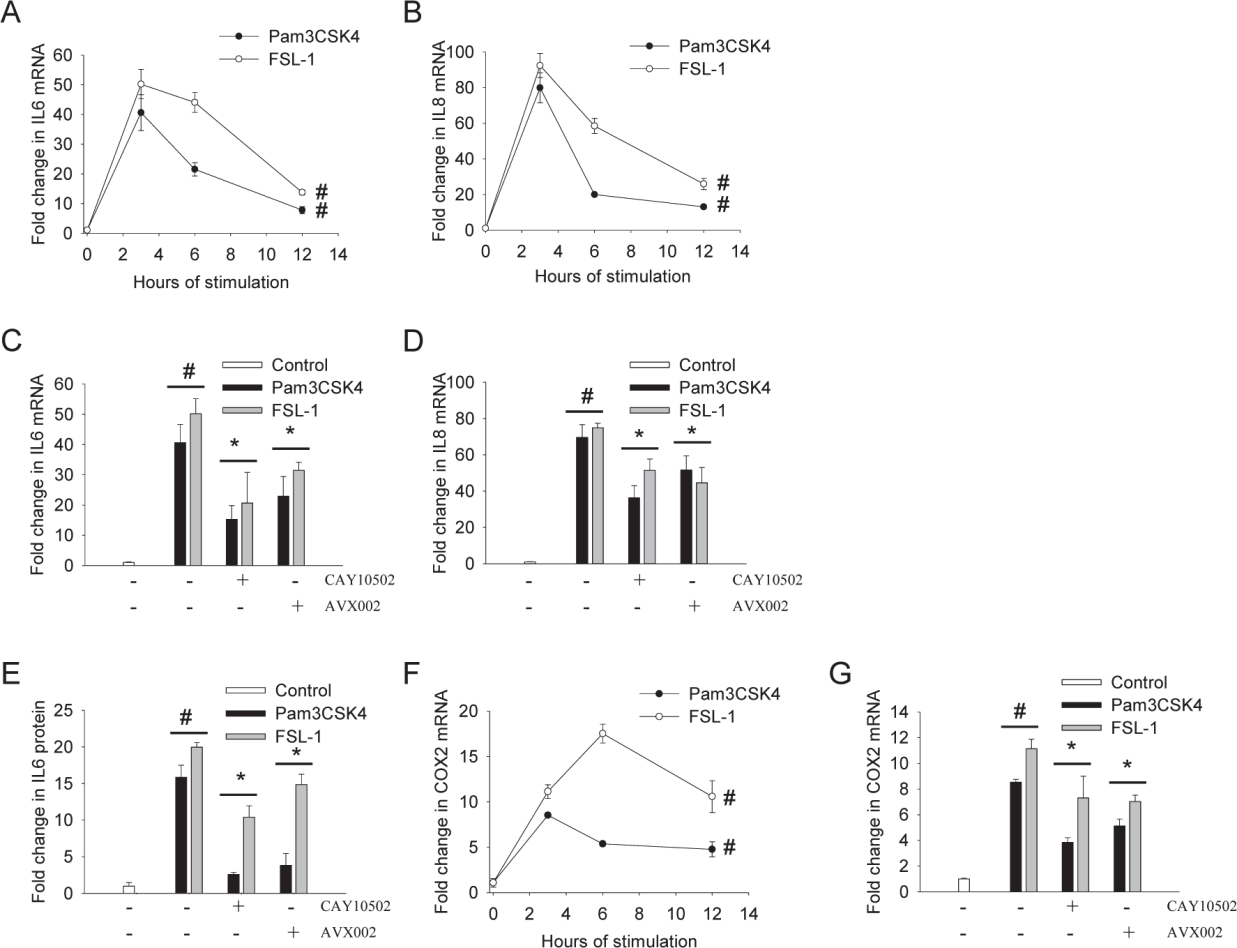
TLR2 ligand-induced gene transcription and protein production involve cPLA2α. Synoviocytes were treated with Pam_3_CSK_4_ (200 ng/mL) or FSL-1 (100ng/mL) for indicated time points (**A**, **B**, **F**), 3 hours (**C**, **D**, **G**) or 24 hours (**E**). Prior to stimulation, cells were pretreated with inhibitors of cPLA2α (5 μM AVX002, 1μM CAY10502, 2 hours). Expression of IL-6 (**A**, **C**), IL-8 (**B**, **D**) or COX2 (**F**, **G**) mRNA was analyzed by qPCR with GAPDH as reference gene, and secreted IL-6 protein (**E**) was analyzed by ELISA as described in the Methods section. Results shown are mean ± SD of at least three independent experiments. Results are considered significant at p ≤ 0.05. Transcription of IL-6, IL-8 and COX2 was found to be significant induced by both ligands at all time points (p^#^ ≤ 0.05) (**A**, **B**, **F**). Significance is indicated by p^#^ ≤ 0.05 when compared to untreated control, and by p* ≤ 0.05 when compared to the respective ligand-treated samples (**C**, **D**, **E**, **G**).

TLR2 activation induce inflammation by upregulating pro-inflammatory genes such as IL-6, IL-8 and COX2 [19], but the role of cPLA2α activity in this pathway is hitherto not well described. Protein levels of the pro-inflammatory interleukin (IL)-6 and IL-8 are both induced by TLR2 ligands in RA synovial cells [44, 45]. By qPCR, we found a time-dependent increase in synoviocyte IL-6 gene transcription in response to Pam_3_CSK_4_ and FSL-1, with maximum induction at 3 hours of 40-fold and 50-fold, respectively (Fig 5A). A stronger induction was detected for IL-8 transcription, which was induced by ~70-fold by both Pam_3_CSK_4_ and FSL-1 after 3 hours (Fig 5B). cPLA2α inhibitors attenuated IL-6 and IL-8 transcription by 40% and 30%, respectively, in response to both Pam_3_CSK_4_ and FSL-1 (Fig 5C and 5D). For IL-6, these results were confirmed at protein level; the detected increase in IL-6 protein levels (16-fold by Pam_3_CSK_4_ and 20-fold by FSL-1) was attenuated by cPLA2α inhibitors by 80–90% in response to Pam_3_CSK_4_ and by 30–50% in response to FSL-1 (Fig 5E). Reduced IL-6 transcription is thus reflected in reduced IL-6 protein production, although the reduction in Pam_3_CSK_4_-induced IL-6 was far more prominent at protein level than at the transcriptional level.

In addition to IL-6 and IL-8, we found that both TLR2-ligands also induced COX2 gene transcription, peaking at 3 hours (Pam_3_CSK_4_) and 6 hours (FSL-1) (Fig 5F). When treated with cPLA2α inhibitors (AVX002 and CAY10502), the TLR2 ligand-induced COX2 mRNA expression was reduced by approximately 50% (Fig 5G). These results suggest a regulatory role of cPLA2α in TLR1/2- and TLR2/6-induced IL-6, IL-8 and COX2 gene expression, and may further indicate involvement of differential post-transcriptional regulating processes of IL-6 protein [46].

### cPLA2α regulates FSL-1-induced IL-6 production through the COX/PGE_2_ pathway

Many of the beneficial pharmacological effects of NSAIDs in RA patients are attributed the inhibition of PGE_2_ synthesis [8]. In the next series of experiments, we investigated if the COX/PGE_2_ pathway is involved in cPLA2α-dependent regulation of gene expression, focusing on FSL-1-induced IL-6 levels.

**Fig 6:**
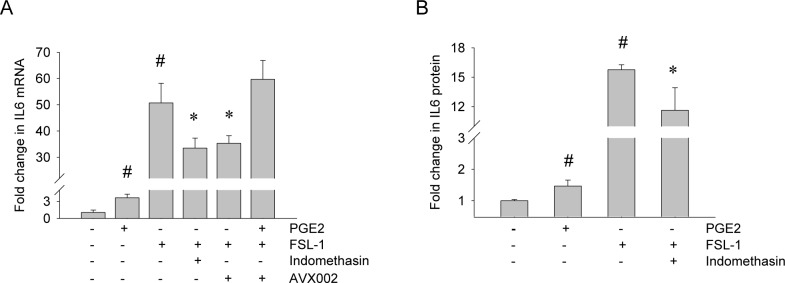
PGE_2_ regulates FSL-1-induced IL-6 production. Synoviocytes were treated with Indomethasin (10 μM, 2 hours) or AVX002 (5 μM, 2 hours) before stimulation with FSL-1 (100ng/mL) and/or PGE_2_ for 3 hours (**A**) or 24 hours (**B**). IL-6 mRNA expression was analyzed by qPCR with GAPDH as reference gene (**A**) and IL-6 protein was analyzed by ELISA (**B**) as described in the Methods section. Results presented are mean ± SD of three independent experiments. Significance is indicated by p^#^ ≤ 0.05 when compared to unstimulated control, by p* ≤ 0.05 when compared to FSL-1-stimulated control

Involvement of the COX enzymatic pathway was investigated using the dual COX1/2 inhibitor Indomethasin. Indomethasin effectively diminished FSL-1-induced PGE_2_ production (Fig 4C), supporting its clinical relevance. Indomethasin reduced FSL-1-induced IL-6 gene transcription by 30% (Fig 6A). This response was again reflected at protein level where Indomethasin reduced FSL-1-induced IL-6 protein by 20% (Fig 6B). The effect of Indomethasin was comparable to that of AVX002 both at mRNA level and at protein level (Fig 5E) indicating that prostanoids produced downstream FSL-1-activated cPLA2α may regulate FSL-1-induced IL-6 production. We next asked whether reduced PGE_2_ levels due to cPLA2α inhibition (Fig 4C) could account for effect of cPLA2α inhibition on IL-6 levels. When added alone, PGE_2_ slightly, but significantly induced both gene transcription and protein production of IL-6 (Fig 6A and 6B). When added in combination with AVX002, PGE_2_ completely rescued the FSL-1-induced IL-6 transcription (Fig 6A). In summary, these results for the first time suggest that PGE_2_ is an important mediator that regulates FSL-1-induced IL-6 production in synoviocytes.

## Discussion

TLR2 signaling is proposed to promote joint destruction and synovitis in RA. In this work, we present for the first time the mechanism in which cPLA2α regulates several important mediators of inflammation in response to TLR2 activation in synoviocytes, namely PGE_2_, COX2, IL6 and IL8.

There is little data available concerning TLR-induced PLA2 activity, AA mobilization and eicosanoid production in RA synoviocytes. In the current work we demonstrate that the TLR2/1 and TLR2/6 ligands Pam_3_CSK_4_ and FSL-1 are potent inducers of synoviocyte cPLA2α phosphorylation, AA release and subsequent PGE_2_ production, in line with responses reported in human leukocytes [25]. Furthermore, cPLA2α inhibition effectively attenuated the TLR-induced AA release and PGE_2_ production, suggesting a central role for cPLA2α in synoviocyte AA metabolism. cPLA2α is expressed in RA synovium and cultured FLS, and its transcription is induced by various pro-inflammatory stimuli, including IL-1β, TNF and lipopolysaccharide [30, 39, 47]. Here, we report that TLR2/1 and TLR2/6 ligands induce synoviocyte cPLA2α gene transcription, indicating that TLR2 ligands activate cPLA2α-dependent AA mobilization directly by increased enzyme activity, and indirectly by transcriptional regulation and *de novo* synthesis as previously described [30].

In various cell types, cPLA2α and sPLA2 act in concert to release AA [43, 48]. In murine macrophages and mast cells, TLR2-induced AA release is dependent on cPLA2α, amplified by GV sPLA2 [26, 35]. However, in IL-1β-stimulated FLS, sPLA2s is not reported to contribute to PGE_2_ production [49]. The latter finding corresponds to our results demonstrating a dominant role for cPLA2α, and minor roles for sPLA2 enzymes in TLR2-induced AA release and PGE_2_ production in synoviocytes. It should however be noted that the sPLA2 inhibitors used in this work are selective for sPLA2 isotypes GIIA, GV and GX, but not GXII [9, 42, 50]. The role of sPLA2 GXIIA is to date unknown, but may withhold important household functions as it is detected at high levels in various tissues [51]. We found that GXIIA was the most highly expressed sPLA2 indicating a hitherto unknown role in synoviocyte cell communication and function, a finding that deserves further investigation. The lack of response to sPLA2 inhibitors in AA release and PGE_2_ production may be explained by a low sPLA2 expression in cultured synoviocytes. RA synovial tissue expresses several sPLA2 isotypes, whereas cultured primary synovial cells only weakly express sPLA2s GIIA, GV and GX transcripts [29]. In agreement with these findings, we report a weak transcriptional sPLA2 expression and a lack of response to sPLA2 inhibitors, suggesting a subordinate role of endogenous sPLA2 enzymes in cultured synoviocytes. Subgroups and splice variants of iPLA2 GVI is expressed in various cells and tissues [52], but their expression and function in the joint is not known. Expression profiles or functional roles of iPLA2 in RA are not previously described; the hereby reported high expression of several iPLA2 subgroups is an interesting finding which indicates a role for these enzymes in the synovium and should be investigated further.

The role of AA metabolites in chronic inflammation and RA disease progression is well established, and reducing PGE_2_ levels by non-steroid anti-inflammatory drugs (NSAIDs) targeting COX enzymes is a well-known strategy for symptom relief and pain management in RA patients [53]. We have previously shown that cPLA2α inhibition normalize AA release, PGE_2_ levels and gene transcription in response to TNF stimulus in synoviocytes [39], a mechanism hereby extended to include FSL-1 and Pam_3_CSK_4_ stimuli. Also, the herein described attenuating effect of cPLA2α inhibition on TLR-induced COX2 gene transcription is similar to our finding in the TNF-response [39], and suggest that cPLA2α inhibitors may target stimuli-induced PGE_2_ production at two levels; directly by reducing AA substrate availability and indirectly by reducing COX2 expression.

Expression levels of several TLRs, including TLR2 and TLR6, but not TLR1, in RA are increased compared to osteoarthritic (OA) synovium [23]. In synoviocytes, we detected transcripts for TLRs 1–7, of which TLR2 was the most abundantly expressed. We further show that TLR expression is regulated by TNF. The TNF-induced up- and down-regulation of TLR2 and TLR4 corresponds to findings in primary synovial fibroblasts from RA and OA joints [24] and support the use of SW982 synoviocytes as a valid *in vitro* model system to mechanistically investigate synovitis. In the present study, FSL-1 was a more potent inducer of cPLA2α phosphorylation and AA release than Pam_3_CSK_4_. This observation is in accordance with findings in human leukocytes [25], and the TLR1:TLR6 expression ratio of 1:12 detected in the current study (Fig 2B). A higher transcriptional expression of TLR6 than TLR1 is also reported in RA synovium [23]. However, post-translational regulatory mechanisms are known to impact TLR protein expression [54]. Caution must thus be taken in comparing transcriptional expression and levels of functional receptors when protein expression data is not available.

TLR2 is shown to regulate the expression of IL-6 in RA FLS [54], and the involvement of PLA2 in this pathway is to our knowledge a novel finding. IL-6 is a pluripotent cytokine involved in pannus formation, osteoclast and FLS activation in RA and plays a key role in chronic inflammation [55]. Increased levels of IL-6 are detected both in the joint and systemically in RA patients [56, 57]. IL-6 deficiency provides protection against development of murine collagen-induced arthritis (CIA) [58, 59], and anti-mouse IL-6 monoclonal antibody suppress CIA development [60]. In humans, anti-IL-6 therapy improves symptoms of RA [61, 62]. Our results show that TLR2-induced synoviocyte IL-6 production is partly controlled by cPLA2α, suggesting an additional beneficial therapeutic effect of cPLA2α inhibitors. The finding that cPLA2α inhibition was more efficient on IL-6 protein than mRNA in response to Pam_3_CSK_4_ than FSL-1 is an interesting finding. IL-6 mRNA stability and degradation are known to be regulated in macrophages by mechanisms including TLR-induced RNase activity [46]. Differential regulation of such post-transcriptional mechanisms may thus influence IL-6 protein levels.

Our results further suggest involvement of PGE_2_ in regulating FSL-1-induced IL-6 expression. PGE_2_ and its receptors are previously shown to regulate IL-1β- and TNF-induced IL-6 generation in human and murine FSL [63–66]. In our experiments, the effect of COX1/2 inhibition was comparable to cPLA2α inhibition in reducing IL-6 production. Furthermore, PGE_2_ resulted in a complete rescue of FSL-1- induced IL-6 transcription following cPLA2α inhibition, suggesting that decreased PGE_2_ levels at least in part account for the attenuating effect of inhibiting cPLA2α activity. This hypothesis is supported by the finding that exogenously added PGE_2_ induced IL-6 expression both at transcriptional and protein levels. Even though PGE_2_-induced IL-6 levels were very low compared to FSL-1-induced IL-6, our results correspond to previously reported results in primary synovial fibroblasts [63] and support a role for PGE_2_ in FSL-1-induced IL-6 production.

The chemokine IL-8 is an important contributor to angiogenesis in the rheumatic joint [67], and acts as a potent neutrophil attractant [68]. IL-8 is associated with the pathogenesis of arthritis, and RA FLS produce IL-8 in both early [69] and established phases of arthritis [70]. cPLA2α has been reported to induce the expression of IL-8 in human lung fibroblasts [71], and is proposed to be a regulator of neutrophil recruitment and inflammation in murine collagen-induced arthritis [72]. This corresponds to our previous findings describing a regulatory role of cPLA2α in TNF-induced IL-8 expression in synovial fibroblasts [39]. Here, we show that cPLA2α also regulates TLR-induced IL-8 expression, suggesting that cPLA2α may act to modulate angiogenesis and neutrophil attraction in synovitis.

In conclusion, our data demonstrate that cPLA2α regulates TLR2-induced lipid biosynthesis and pro-inflammatory gene expression in synoviocytes, in part through the COX/PGE_2_ pathway. Seen in context with our previous finding that cPLA2α also regulates TNF-induced signaling [39], these results expand our understanding of cellular signaling mechanisms modulated by cPLA2α activity and indicate a central regulatory role for cPLA2α in synovitis.
